# Trailblazing Person‐Centred Care: Lessons From a Hospital Cluster‐Wide Retreat to Co‐Design Approaches to Person‐Centred Care in Singapore

**DOI:** 10.1111/hex.70594

**Published:** 2026-04-25

**Authors:** Xiankun Meng, Amanda Wei Li Tan, Samantha Shi Man Koh, Shanice Shi Hui Ng, Balkhis Puteh, Chien Earn Lee, Esther Li Ping Lim, Luke Sher Guan Low, Stephanie Swee Hong Teo, Andy Gim Hong Sim

**Affiliations:** ^1^ SingHealth Centre for Person‐Centred Care Singapore; ^2^ ESTHER Network Singapore Singapore; ^3^ Regional Health System SingHealth Singapore; ^4^ Allied Health Division Singapore General Hospital Singapore; ^5^ Sengkang Community Hospital Singapore; ^6^ Group Nursing, SingHealth Singapore

**Keywords:** co‐design, implementation science, organisational change, Person‐centred care, shared responsibility

## Abstract

**Introduction:**

Person‐centred care (PCC) is globally recognised as essential for quality healthcare, yet implementation remains inconsistent in Singapore due to hierarchical structures, time constraints, and fragmented understanding. SingHealth, Singapore's largest public healthcare cluster, initiated a retreat to co‐design a unified approach to PCC across its institutions.

**Objectives:**

The retreat aimed to (1) align stakeholders on PCC's importance; (2) co‐develop a locally tailored PCC definition; (3) advance PCC through five workstreams (user experience, research, education, service innovation, strategic partnerships); and (4) provide experiential co‐design learning.

**Methods:**

This qualitative case study employed reflexive thematic analysis to analyse data from a retreat designed using Goffman's frame analysis. The retreat engaged 42 healthcare practitioners, 26 senior management, and 13 patient/caregiver experience experts in a three‐phase co‐design process.

1. Pre‐design: Strategic stakeholder invitation and pre‐reading to align perspectives.

2. Generative phase: Creative methods (‘Frozen’ metaphors, flip cube exercises and patient videos) to explore PCC's ‘what’ and ‘why’, followed by live visual scribing, discussion and presentation.

3. Evaluative phase: Visioning exercises, debates on patient autonomy, and development of an implementation matrix.

**Results:**

Four key themes emerged:.

1. Shared responsibility between patients and providers.

2. Communication skills as foundational to PCC.

3. Integration of PCC into systems and culture.

4. Actionable steps, including patient‐reported experience measures and new models of care.

Tangible outputs included a shared vision‐ ‘Empowering individuals. Everyone matters’ and a five‐level implementation matrix operationalising PCC at institutional, departmental, and patient‐provider levels.

**Conclusion:**

The retreat successfully fostered stakeholder alignment and co‐designed actionable strategies for PCC. The co‐design process successfully revealed nuanced tensions around shared responsibility while generating implementable strategies. This model demonstrates how structured stakeholder engagement can advance PCC in hierarchical healthcare systems.

**Patient or Public Contribution:**

Experience experts (patients and caregivers), constituting 16% of retreat participants, were strategically recruited from established networks. They actively co‐designed the PCC definition, participated in world café discussions, informed implementation strategies across five workstreams, co‐created the vision statement and validated the final implementation matrix. Their lived experiences critically shaped key themes of this event. One experience expert co‐authored this manuscript, reflecting sustained partnership.

**Abbreviations**
CPCC: SingHealth Centre for Person‐Centred Care
HCP: healthcare professional
HER: electronic health record
PCC: person‐centred care
PREMs: patient‐reported experience measures

## 1 Introduction

Over the past decade, person‐centred care (PCC) has gained considerable traction within the healthcare sphere [1]. Both healthcare professionals (HCPs) and management are increasingly recognising the need to reexamine the relationship between individuals and providers. Conventional disease‐centric biomedical model has primarily focused on the biological deficiencies causing illness, leading to a directive and expert‐led approach to care. This model positions doctors and other HCPs as experts who determine the most appropriate treatment options and strategies for managing medical conditions, expecting patients to comply without question [2]. However, the biomedical model has been criticised for being overly reductionistic [3].

In the mid‐20th century, George Engel introduced the biopsychosocial model, challenging the reductionism of the biomedical paradigm [4]. By emphasising the interplay of biological, psychological, and social dimensions, Engel laid the groundwork for PCC. This holistic perspective views patients as individuals with unique needs and preferences rather than as passive recipients of disease‐focused treatment. Concurrently, societal shifts towards patient empowerment – especially among younger and more educated populations – have accelerated the demand for shared decision‐making in healthcare [5, 6, 7].

Despite its global significance, PCC is not yet a societal norm in Singapore's healthcare system. A study by Lee et al. demonstrated that Singaporean undergraduate medical students are less likely to treat their patients as equal partners compared to their US counterparts [8]. Additional local studies further highlight the gap between the aspiration and practice of PCC. For instance, Nurjono et al. found that while healthcare providers in Singapore acknowledge the value of PCC, its implementation remains inconsistent due to system‐level barriers, hierarchical care structures, and time constraints [9]. Similarly, Lim et al. noted that while patients express a desire for more collaborative care, healthcare delivery remains predominantly provider‐driven [10].

Singapore's aging population and increasing prevalence of chronic diseases [11] demand care models that prioritise long‐term partnerships between patients and providers. However, current studies reveal a mismatch between patients’ expectations and providers’ readiness. International bodies like the World Health Organization now mandate PCC as a cornerstone of quality care [1]. Singapore's vision to empower residents to take charge of their own health [11] cannot be realised without addressing gaps in PCC implementation. Thus, PCC is not just an aspirational goal but a *critical imperative* for modern healthcare systems.

Global and local backdrops underscore the need for deliberate efforts to translate PCC ideals into practice, making SingHealth's initiative particularly trailblazing in the local context. SingHealth, Singapore's largest public healthcare cluster, is deeply committed to its common purpose: ‘Patients. At the Heart of All We Do’. This commitment is exemplified by the establishment of the Centre for Person‐Centred Care (CPCC). Through the CPCC, SingHealth is advancing its goal of embedding PCC into everyday practice, fostering truly person‐centred outcomes. This paper presents a qualitative case study of SingHealth's cluster‐wide retreat, using reflexive thematic analysis to identify key themes, barriers, and facilitators in co‐designing approaches to PCC. This study is reported in accordance with the Consolidated Criteria for Reporting Qualitative Research (COREQ) checklist (see Supplementary File).

## 2 Objectives

The retreat was designed to address the fragmented understanding and inconsistent practice of PCC across SingHealth institutions. It served as a pivotal platform to convene diverse stakeholders – including healthcare professionals, administrators, and patient experience experts – to co‐design a shared vision, develop a locally tailored definition of PCC, and identify actionable strategies for implementation. The objectives of the retreat were fourfold:

1. Align on the importance of PCC: Establish a shared understanding of the significance of PCC within the organisation.
2. Adopt a locally tailored definition of PCC: Co‐develop a definition of PCC that reflects the organisation's unique context and specific needs.
3. Advance PCC through five key implementation workstreams – user experience, research, education, service innovation and translation, and strategic partnerships.
4. Provide an experiential understanding of co‐design: Immerse participants (service providers and users) in the co‐design process, a rare but valuable approach in Singapore's healthcare setting.

## 3 Methods

### 3.1 Study Design

This study is a qualitative case study of a cluster‐wide retreat. The retreat was designed and facilitated using Goffman's frame analysis as a guiding conceptual framework, with activities intentionally structured to shift participants’ frames of reference, as detailed in Table 1. It is critical to distinguish the design framework from the analytical method—while Goffman's framework guided the intervention's structure, the qualitative data generated were analysed using Braun and Clarke's reflexive thematic analysis [12].

**Table 1:** Mapping of retreat programme flow to goffman's frame analysis.

Stages of co‐design	Activities	Purpose as per Goffman's frame analysis
**Pre‐design**	Strategic invitation of retreat participants	*Frame alignment*: Ensure participants share a primary framework by inviting diverse stakeholders (HCPs, administrators, patients) to establish a common interpretive lens for PCC.
	Communication of the agenda and selection of pre‐reading materials	*Prevent frame misalignment*: Use pre‐readings to prime participants for the social framework of PCC, reducing clashes between pre‐existing and intended frames.
**Generative**	The Frozen Movie Theme Metaphor	*Frame setting via keying*: Use metaphors (‘Let It Go’ and ‘Into the Unknown’) to key the retreat's activities, transforming paternalistic care into a PCC frame.
	The Energiser Activities	*Address frame breaks via fabrication*: Disrupt paternalistic frames using flip cubes and patient videos, intentionally reshaping perspectives.
	Deep Dive into Two Framing Questions in world cafe	*Reinforce the new frame*: Group discussions ‘*What is PCC to our organisation*’ and ‘W*hy is PCC important*’ solidify the social framework through examination of shared experiences.
	Integrating ‘Live’ Visual Artwork	*Frame anchoring*: Visual scribing externalises abstract discussions into a shared artefact, stabilising the PCC frame as a tangible reference.
	From Ideas to Action Discussions and Presentations	*Frame stabilisation*: Rapid prototyping with questions operationalises the PCC frame into workflows, embedding it into five PCC workstreams.
**Evaluative**	Dissemination of retreat outputs	*Frame expansion*: Propagate the PCC frame organisation‐wide, extending its social framework beyond the retreat context.
	Visioning exercise	*Frame codification*: Co‐create a vision statement (‘Empowering individuals. Everyone matters’.) to formalise the PCC frame as an organisational primary framework.
	Debate	*Frame testing*: Surface tensions (e.g. patient autonomy vs. clinical expertise) to assess the PCC frame's resilience and adaptability.
	Development of implementation matrix	*Frame institutionalisation*: Translate co‐designed strategies into an implementation matrix, embedding the PCC frame into institutional practices.

### 3.2 Ethical Considerations

This retreat was conducted as a quality improvement initiative to develop organisational strategy. Per institutional policy, such internal quality improvement activities focusing on service development do not require formal Institutional Review Board review. All participants were informed of the purpose of the retreat and consented to contribute to group activities and outputs. Consent was also obtained from one of the co‐authors of this paper, Ms Balkhis Puteh [B.P.], who was also a participant‐experience expert of the retreat, for her reflections and quotations to be included in this manuscript.

### 3.3 Participant Selection and Sampling

A purposeful sampling strategy was employed to ensure the inclusion of key stakeholders with direct experience or influence in PCC implementation within the hospital‐cluster. Participants were selected based on the following criteria:

Inclusion Criteria: (1) HCPs (doctors, nurses, allied health) actively involved in PCC initiatives; (2) Senior management with decision‐making authority to drive organisational change; (3) Patient and caregiver experience experts from established networks (SingHealth Patient Advocacy Network, SPAN and ESTHER Network Singapore) with lived experience of the healthcare system. ESTHER Network, a PCC innovation model from Sweden, was adopted in Singapore to drive the philosophy of PCC and improve integration between health and social care practitioners through improvement projects [13].

Exclusion Criteria: Individuals without a direct role or stake in PCC implementation within the cluster.

The retreat engaged a purposeful sample of 81 participants. Sample size was determined by the principle of information power [14], which posits that the required number of participants depends on the study's potential information yield. Our study was designed with strong inherent information power—narrow, well‐defined aims; a highly specific sample of experts and stakeholders central to PCC implementation; and a design that generated a rich, multi‐modal dataset (written, visual, verbal). Consequently, this sample size was deemed sufficient to provide the depth and breadth of data required to address our research objectives. In line with common qualitative reporting, our analysis had achieved thematic sufficiency, as after extensive engagement with the data, we were generating rich, nuanced interpretations rather than substantively new thematic concepts.

**Figure 1:**
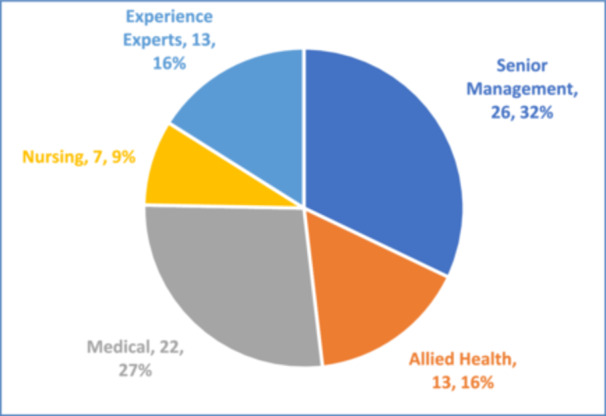
Breakdown of retreat participants. *Note:* Management includes Communications, Institute of Patient Safety and Quality, Office of Value Based Care, Population Health, Staff Wellness, Future Workforce and Operations.

The final group comprised 52% healthcare practitioners, 32% senior management, and 16% patient/caregiver experience experts (Figure 1). Experience experts brought invaluable insights from their personal healthcare journeys, ensuring that the co‐creation process was grounded in authentic users’ perspectives. B.P., one of the participating experience experts who also agreed to co‐author the paper, reflected on her experience, ‘First of all I feel that my involvement in the event is important to me because as a patient and a caregiver myself, I am able to position on both sides of the spectrum… This engagement broadened my own understanding of the direction of person‐centred care’.

### 3.4 The Retreat and Data Collection

The retreat was structured as a three‐phase co‐design process: pre‐design, generative, and evaluative. Multiple sources of qualitative data were collected during these phases to ensure a rich and comprehensive dataset:

Written materials: Flip‐chart notes generated during world café sessions and group discussions. Entries submitted for visioning exercise.

Visual artefacts: The live‐scribed digital artwork and the final implementation matrix.

Field notes: Notes taken by facilitators during the energiser activities, presentations and debate.

This multi‐faceted data collection approach provided a robust foundation for subsequent qualitative analysis and allowed for triangulation.

### 3.5 Data Analytical Approach

Following the principles of reflexive thematic analysis, we explicitly position our analysis within a constructionist epistemology, viewing meaning and experience as socially produced within the retreat context. Our orientation was primarily experiential, aiming to understand and prioritise participants’ accounts of their attitudes and experiences regarding PCC. The analysis was inductive (themes generated from the data), though informed by our broad research objectives. We engaged in latent coding, looking beyond the semantic content of the data to interpret underlying assumptions, tensions, and ideas about power, responsibility, and system change.

The data were analysed using the six‐phase reflexive thematic analysis process outlined by Braun and Clarke [12]. This method was selected for its flexibility and suitability for identifying patterns of meaning across a complex dataset. The analysis was conducted collaboratively by two researchers (X.M. and S.K.), who documented their reflexive codes throughout.

Familiarisation: Researchers independently immersed themselves in the full dataset (written notes, visual artefacts, field notes), noting initial observations.

Generating Initial Codes: Using Microsoft Word, we systematically applied inductive, latent codes to data from different sources. The iterative coding process was recorded separately on Microsoft Excel.

Generating Themes: Codes were collated and sorted into themes through iterative discussion.

Reviewing Themes: Themes were checked against the coded extracts and the entire dataset to ensure they formed a coherent pattern.

Defining and Naming Themes: The essence of each theme was articulated, and clear definitions and names were generated for each.

Producing the Report: The analysis was woven into a narrative account, supported by vivid data extracts.

### 3.6 Data Integration and Validation

To ensure a coherent analysis, data from different sources were integrated throughout the coding process. For example, a concept emerging from world café notes (shared responsibility) was cross‐referenced with visual scribe artwork, which depicted ‘joint responsibilities’ and facilitator field notes from the debate. This triangulation of sources strengthened the validity of the codes and ensuing themes by confirming that patterns of meaning appeared across different types of data and participant interactions.

The analysis was validated through several means: (1) investigator triangulation via collaborative analysis by two researchers; (2) member checking of key outputs (the implementation matrix and vision statement) with participants after the retreat; and (3) a reflexive dialogue between coders to challenge interpretations and ensure they were grounded in the dataset.

### 3.7 Coder Reflexivity and Handling of Disagreements

Both researchers journaled in reflexivity memos, acknowledging their positions as healthcare insiders —X.M. as a former pharmacist and S.K. as a physiotherapist—which informed their sensitivity to practical barriers and systemic tensions in PCC practice. Disagreements in coding or thematic interpretation were not resolved by seeking consensus on a single ‘correct’ code. Instead, divergent interpretations were explored as reflective of our distinct analytical lenses and the multifaceted nature of the data. These discussions deepened the analysis, with divergent interpretations often revealing higher‐order conceptual tensions that were instrumental in theme development (e.g., the evolution of theme 1 from ‘shared decision‐making’ to ‘shared responsibility’). Thus. the final thematic structure represents a synthesised interpretation born from this critical, collaborative dialogue.

## 4 Description of Retreat

The retreat was carefully structured to align with its primary objectives, following three out of four distinct stages of co‐design: pre‐design, generative and evaluative phases [15].

### 4.1 Pre‐Design

Central to the effective implementation of PCC is the concept of co‐creation. In a healthcare context, co‐creation refers to the collaborative partnership between patients and healthcare professionals, working together to co‐deliver care that is personalised to the patient. It positions patients as active partners in co‐producing their healthcare services, fostering mutual respect and shared responsibility [16]. Within the PCC framework, co‐creation emphasises the systematic inclusion of patient participation in the development process, recognising their expertise and active involvement [17]. This interaction between healthcare professionals and patients leads to value creation for patients, in contrast to the traditional view where the healthcare system solely creates the value that the patient receives [18].

### 4.2 Communication of the Agenda and Selection of Pre‐Reading Materials

Communication of the agenda, selection of pre‐reading materials, and the conduct of the retreat itself are guided by Goffman's frame analysis [19]. According to Goffman, the concept of frame refers to ‘the principles of organisation that govern events and our subjective involvement in them’, essentially serving as a contextual point of reference that shapes how individuals interpret experiences [20]. Thus, frame analysis investigates various contexts and examine how these contexts influence participants in constructing meaning collectively from their experience. We adopt frame analysis in designing our retreat because we believe a controlled context setting and the maintenance of an appropriate context are crucial to the co‐design process.

Cognisant of the reality that PCC was not the societal norm, where an agreed‐upon definition of PCC did not exist, there was an inherent risk of frame misalignment if the participants did not see the importance of PCC, or if they did not believe they were able to make the transition from paternalistic care to person‐centred care. Frame misalignment might result in impasses in the co‐designing process.

To align participants on the context of the retreat, all confirmed attendees were sent the journal paper ‘*Person‐Centred Care − Ready for Prime Time*‘ [21], to introduce the conceptual shift from patient to person‐centred care. This served as a foundation for frame alignment. The retreat confirmation emails also detailed the retreat agenda, emphasising the primary discussion frames.

### 4.3 Generative Phase

The generative phase started with two energiser activities. Four primary framing questions were written to generate responses from the participants:

1. What is PCC to our organisation?
2. Why is PCC important?
3. How are we going to advance PCC?
4. How do we know we are in the right direction?

The first two were discussed in a world café format, with a ‘live’ visual artist transcribing learning into a piece of collective artwork. The next two were examined by retreat participants in five implementation domains and expounded in group presentations.

### 4.4 Weaving Creativity into Co‐Design: The Frozen Metaphor

To enhance engagement and foster creative thinking during the generative phase, the retreat creatively integrated themes from Disney's animation movie *Frozen*, leveraging the familiar narratives of ‘*Let It Go*’ and ‘*Into the Unknown*’ to frame the discussions. The ‘*Let It Go*’ theme corresponded to the world café, where participants discussed ‘*What is PCC to our organisation?*’ and ‘*Why is PCC important?*’ This phase encouraged participants to release preconceived notions of traditional, provider‐led care and embrace new perspectives aligned with person‐centred principles.

Building on this momentum, the ‘*Into the Unknown*’ theme guided the subsequent discussions around ‘*How are we going to advance PCC?*’ and ‘*How do we know we are in the right direction?*’ This framing encouraged participants to explore uncharted territories of healthcare innovation with openness and curiosity. Using metaphors not only made the co‐design process more engaging but also facilitated deeper emotional connections, which are known to enhance creativity and collaborative knowledge building [22]. This playful yet purposeful approach ensured that participants remained energised, open‐minded, and fully engaged throughout the retreat.

### 4.5 The Energiser Activities

The retreat integrated two energiser activities—‘serious play’ and an experience expert interview clip—designed as probes to orient participants toward common primary frames while addressing potential frame breaks. Frame breaks can occur when participants’ pre‐existing beliefs conflict with the intended primary frame, hindering progress in the co‐design process. To mitigate this, the retreat employed the process of fabrication [19], a deliberate yet benign alteration of frames to reshape participants’ perspectives.

**Figure 2:**
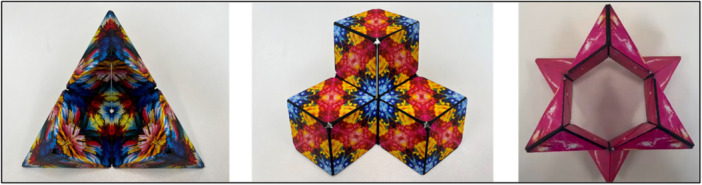
Various shapes of flip cubes in isolation or combined.

**Figure 3:**
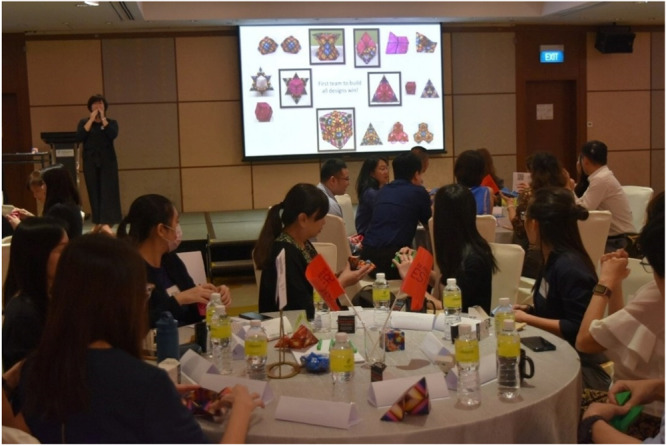
Retreat participants were manipulating flip cubes.

Firstly, a ‘serious play’ activity using flip cubes was conducted. Flip cubes are toys that can be expanded or rotated to form various geometrical shapes either in isolation or in combination (Figure 2). Participants were required to manipulate the cubes to achieve specific shapes using a combination of cubes within their tables (Figure 3). This activity served two purposes:

1. Learning in Affective Domains: Holiday and colleagues posited that the tactile nature of serious play activities can bring tacit knowledge to the surface by drawing on perceptual dimensions of experience [23]. Similarly, we drew a parallel lesson between flip cube play and Lewin's Change Model [24], which showed the transformation from apparent equilibrium to the state of unfreeze, change, and refreeze. The participants’ reflection from their serious play, coupled with Lewin's Change Model, implicitly ‘un‐froze’ the paternalistic frame and shifted the frame towards that of PCC.
2. Relationship Building and Fostering Inclusiveness: Establishing a shared, challenging goal through the flip cube activity promoted communication and collaboration among the retreat participants. This form of serious play encouraged interaction among individuals who typically do not collaborate, breaking down professional silos and facilitating open dialogue [25]. The novelty of the flip cubes further reduced power hierarchies, creating an inclusive environment where all participants felt equally empowered to contribute. Research has demonstrated the positive impact of game‐based learning on social inclusion, particularly among school‐aged children [26, 27]. This principle was equally effective in the adult setting, as the playful activity stimulated engagement, enhanced mutual understanding, and reinforced the collaborative spirit.

**Figure 4:**
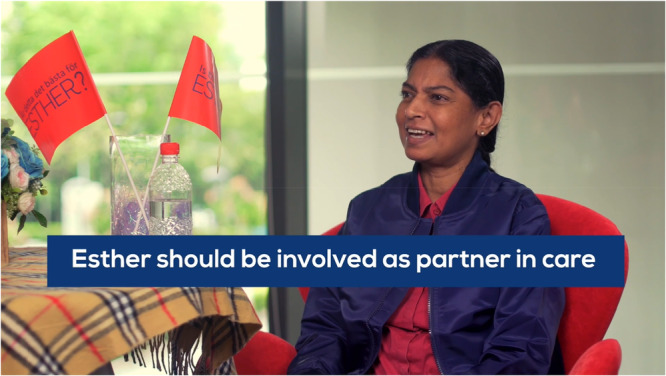
Interview clip with the experience expert.

Secondly, a short interview clip was featured to challenge participants’ perceptions of paternalistic care (Figure 4). In the video, a patient recounted her encounter with a paternalistic physician who disempowered her desire to seek self‐care tips. The video concluded with the patient wishing for the healthcare team to involve her potential as a useful resource, instead of restricting her role as a passive participant in healthcare.

Short films are effective in focusing stakeholders’ attention on key issues in experience‐based co‐design [28]. This clip served as a value probe, prompting participants to reflect on their own values and prepare for value challenges in the ensuing discussion activities. The film clip also readied the participants for the frame transition, where the level of learning shifted from receiving to responding as per Krathwohl's affective domains [29].

### 4.6 Deep Dive into Two Framing Questions: What and Why

Group discussion was the tool employed to explore the first two primary framing questions. World café, a rotational discussion format, facilitated diverse perspectives. The world café activity was designed to maximise participation, promote diverse perspectives, and ensure inclusive dialogue around key questions.

There were two rounds of world café discussions, each focused on one of two questions. Participants were evenly allocated into ten discussion groups for the initial round of world café discussions, with five groups focused on the question ‘*What is PCC to our organisation?*’ and the other five exploring ‘*Why is PCC important?*’ Each group was deliberately composed to ensure a balanced representation of healthcare professionals, senior management, and experience experts. The inclusion of at least one experience expert per group ensured that the discussions were grounded in lived experiences of PCC within the healthcare system. After 15 min, participants rotated to a new table to deliberate on the other question. This deliberate rotation ensured cross‐pollination of ideas while maintaining an even mix of participants.

### 4.7 Integrating ‘Live’ Visual Artwork to Boost Learning of Ideas

**Figure 5:**
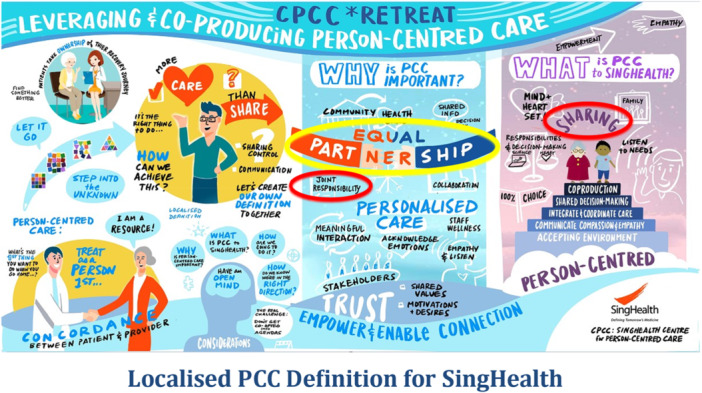
Visual artwork of the first two framing questions.

Art‐based activities fostered knowledge sharing by transforming abstract concepts into tangible, visual representations, making it easier for participants to communicate and consolidate ideas [30]. During the world café discussions, participants contributed their insights and reflections, which were synthesised by facilitators and scribes. These key points were then conveyed to a professional visual scribe, who created live digital illustrations in real‐time (Figure 5). This visual documentation not only captured the diversity of perspectives but also highlighted connections between ideas that might have been overlooked in verbal exchanges. By externalising the group's collective thinking into a shared visual artefact, abstract discussions on what and why PCC matters were transformed into an accessible, holistic representation that resonated with all participants. Visual scribing additionally facilitated frame transition by establishing a common basis for ensuing discussions.

To ensure alignment with the retreat's objectives, a professional visual artist was engaged prior to the event. Several preparatory discussions were conducted to communicate expectations, clarify the retreat's key themes, and establish how visual representation could enhance knowledge synthesis.

### 4.8 From Ideas to Action

Following the world café, participants were reassigned to new discussion tables based on their professional background and area of expertise. These tables were organised around five key implementation workstreams: user experience, research, education, service innovation and translation, and strategic partnership. This deliberate reassignment ensured that participants could contribute meaningfully to discussions aligned with their expertise while continuing to engage with diverse perspectives. During this round, all groups explored two critical questions: ‘How are we going to advance PCC?’ and ‘How do we know we are in the right direction?’ These two questions encouraged participants to conceptualise actionable strategies while considering measurable indicators for success.

Each table was facilitated by a trained domain lead who facilitated the conversation and ensured balanced contributions among participants. A dedicated scribe at each table captured key insights in real‐time, enabling the consolidation of discussion points for the next phase. This structured approach not only optimised participation but also fostered a collaborative atmosphere, ensuring that diverse perspectives were heard, valued, and integrated into the co‐designed outcomes.

To facilitate idea generation and refinement, participants engaged in rapid prototyping, sketching out preliminary concepts, workflows, and strategies that could be tested and iterated upon. This hands‐on approach not only fostered creativity but also ensured that proposed initiatives were both practical and aligned with the organisation's context.

Adding a creative touch, the Frozen theme ‘*Into the Unknown*’ was woven into the presentation phase. Each group was invited to present their implementation strategies as if embarking on an exploratory journey, symbolising the healthcare system's shift toward PCC. This playful yet purposeful framing energised the participants and reinforced the mindset of embracing innovation while navigating uncharted territories.

The discussions culminated in five group presentations, with each workstream presenting two sets of strategies—one focused on implementation and the other on evaluation. These presentations not only showcased the participants’ collective insights but also served as the foundation for the retreat's final deliverable: a cluster‐wide PCC implementation matrix.

### 4.9 Evaluative Phase

Drawing from the visual art and presentations generated from the retreat, the evaluative phase was designed to engage a wider population of healthcare professionals and experience experts. The discussion points and visual art were disseminated to a wider organisational audience through email and a townhall event.

A visioning exercise was held where participants of the retreat as well as staff members from all SingHealth institutions were invited to co‐create a vision statement with CPCC. A call was made for submissions. Out of 72 entries, five were shortlisted for voting. The final statement ‘*Empowering individuals. Everyone matters*’. was chosen as it highlighted the importance of everyone, not just patients and their families, but also healthcare practitioners. The important element of empowerment was something that many resonated with – given that a central component of PCC was in empowering patients to achieve what mattered to them. Similarly, the vision is for healthcare practitioners to be empowered and supported to deliver care in a way which is meaningful to them, by helping patients achieve their goals.

The evaluative phase culminated with a debate on the topic ‘patients should be given more power to make decisions about their health’. This debate was structured to explore the implications and challenges of shifting decision‐making power from healthcare providers to patients. Participants were engaged in discussions to clarify how increased patient autonomy could be practically implemented within the healthcare system, while addressing potential concerns such as the need for balanced information provision and the role of healthcare providers in guiding patient decisions. The insights from this debate informed the development of the implementation matrix (Table 2), which facilitates the generation of actionable ideas at patient‐provider, departmental and institutional levels.

**Table 2:** SingHealth CPCC implementation matrix developed.

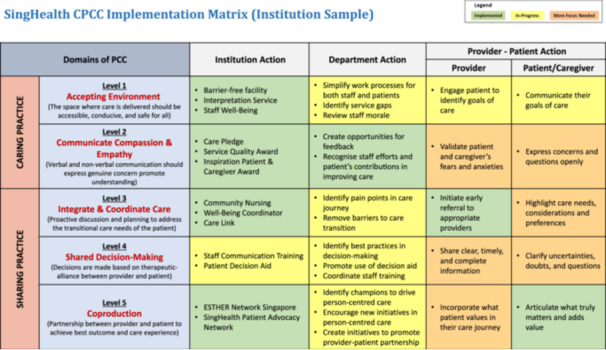

## 5 Results

Our reflexive thematic analysis of the multi‐source qualitative data identified four central themes regarding the implementation of PCC. These themes represent the core patterns of meaning that emerged from our collaborative interpretation of participant discussions, reflections, and co‐design outputs.

### 5.1 Theme 1: From Shared Decision‐Making to Shared Responsibility

The analysis revealed that participants conceptualised PCC not as a simple transfer of decision‐making authority, but as a fundamental rethinking of power dynamics requiring shared responsibility.

This nuanced view was evident in the debate on patient autonomy, where HCPs expressed concerns about moral responsibility, with one asking, ‘If the patient makes a choice against medical advice, who is ultimately responsible for the outcome?’ Similarly, patient experience experts in world café discussions highlighted the burden of decision‐making, with one stating, ‘I don't want to be abandoned with all the decisions—I need my doctor's expert knowledge and guidance’. These contrasting perspectives reveal that shared responsibility involves navigating the clinician's duty of care alongside the patient's need for supported autonomy, not simply handing over choice.

This vision of partnership was captured in a Strategic Partnership workstream note: ‘What makes a person‐centred doctor? It should be a mutualistic approach, whereby both patients and doctors are involved deeply in patient care’. The term ‘mutualistic’ explicitly reframes the relationship as one of interdependent roles and shared investment in the outcome.

The tension between autonomy and responsibility was physically enacted in the flip cube debrief. Participants reflected that the challenge of building a new structure directly paralleled the clinical dilemma—both required negotiating individual initiative with collective accountability to achieve a shared goal. This collective accountability was visually captured in the live scribing artwork as ‘joint responsibilities’.

### 5.2 Theme 2: Communication As a Foundational Skill

Communication was constructed not merely as a technique, but as the essential bedrock for building empathy, trust, and effective partnerships. Participants defined it as the primary mechanism for demonstrating respect and establishing the human connection necessary for all other aspects of PCC.

The critical importance of this theme was powerfully underscored by the patient video clip, where the experience expert emphasised feeling like a passive recipient and wished to be seen as ‘a partner in my own care’. World Café notes explicitly defined the desired shift—‘Patient wants to be heard, understood, listened, respected’ and ‘Putting aside assumptions focus on the person instead of the disease; listen to the patient’. These statements show that participants viewed effective communication as an active, person‐first process of validation, which is prerequisite to trust and collaboration.

This sentiment was echoed throughout the world café notes, which repeatedly referenced ‘active listening’ and ‘understanding patient priorities’, with multiple groups independently identifying ‘empathy’ and ‘trust’ as direct outcomes of effective communication. The live scribe artwork visually anchored this concept, featuring phrases like ‘listen to needs’ and ‘meaningful interaction’ prominently.

The foundational role of communication was summarised by a HCP during workstream presentations, who noted, ‘We're trained to diagnose and treat, but we're not systematically trained to listen in a way that makes patients feel understood’. This reflection highlights a perceived systemic gap in training, positioning communication not as a natural trait but as a core clinical skill that must be developed.

A concrete proposal from the Service Innovation workstream aimed to measure this— ‘Getting feedback on patient experience… ‘How often did the doctors explain things in a way you could understand, listen carefully to you, and treat you with courtesy and respect?’’ By proposing to measure these specific communicative behaviours, participants framed them as accountable, improvable practices integral to quality care.

### 5.3 Theme 3: Systemic Integration As a Prerequisite for Sustainable Change

A strong consensus emerged that individual HCP's efforts are insufficient without supportive systems and a reinforcing culture. Participants consistently highlighted how current structures often hinder person‐centred practices, framing system change not as a luxury but as a necessity.

Workstream discussions on service innovation identified specific systemic barriers, with one group noting our current healthcare system often reflects fragmented care delivery, which hampers the seamless coordination of services. Without systems designed to facilitate care coordination, it is challenging for staff to deliver a cohesive care plan. Another group identified a specific systemic barrier, noting, ‘Our electronic health records are designed for documentation, not for facilitating conversations about patient goals’.

Critically, participants did not just identify barriers. They proposed integrated, structural solutions that would redistribute responsibility and create new enabling systems. For example, one proposal called to ‘Redesign system workflow to be patient centric’, moving the locus of change from the clinician to the care process itself. Another explicitly linked a new systemic role to sustainable care coordination – ‘Having a Case Manager for each patient… dedicated Case Managers who can be part of the MDT (Multi‐Disciplinary Team) and act as advocates for patients in the whole continuum of care’. These suggestions demonstrate a clear understanding that sustainable PCC requires embedding support into the organisational architecture – creating new roles and redesigning processes – rather than relying on individual clinicians to overcome inadequate systems.

This was compounded by structural issues, as a senior leader argued during the evaluative debate, ‘We can train our staff in communication skills, but if we don't give them the time in their schedules or change our performance metrics, we are setting them up for failure’. This quote powerfully encapsulates the theme, because training, as an individual‐level intervention, is ineffective without systemic enablers.

The direct response to this theme was the creation of the implementation matrix, which operationalised this systems‐thinking by including care integration strategies at institutional and departmental levels, such as ‘Well‐Being Coordinator’ (trained lay‐persons to support HCPs in provision of PCC on behavioural changes) and ‘remove barriers to care transition’.

### 5.4 Theme 4: The Imperative for Actionable Steps and Measurable Outcomes

The analysis confirmed a clear demand to translate the philosophy of PCC into concrete behaviours and to evaluate progress with relevant metrics. Participants expressed frustration with abstract ideals and sought clarity on what to do differently and how to know if it works, revealing a pragmatic drive for implementation.

This need was voiced by a HCP seeking specificity, ‘Tell me what to do differently at the bedside. ‘Be more person‐centred’ is too vague’. This plea underscores the theme by highlighting that goodwill is not enough; staff require clear, behaviourally‐defined scripts. In response, discussions generated granular, actionable steps. A User Experience workstream suggestion provided one such script: ‘Start by asking patients what outcome they hope for, what they are willing to commit to, and what's important to them’. Such questions transform the principle of partnership into a replicable clinical action.

Furthermore, the demand for measurement was unequivocal. The Research workstream stated the need for ‘More disease areas including PREMs/PROMs in measurements’, highlighting the need to expand the routine collection of patient‐reported data beyond a few select conditions. Furthermore, the User Experience workstream argued, ‘What gets measured, gets managed… If we only emphasise clinical outcomes in key performance indexes, we signal that patient experience matters less’. These statements directly link measurement to values, arguing that without quantifiable patient‐experience metrics, PCC remains a secondary, optional concern.

The visioning exercise, which culminated in the statement ‘Empowering individuals. Everyone matters’, served as a foundational step in creating a clear and actionable guiding principle for the entire organisation.

Finally, the co‐creation of the five‐level implementation matrix was the ultimate tangible output of this theme, answering the demand for specificity by defining who (Institution, Department, Provider/Patient) needs to do what (concrete actions) across the domains of PCC.

## 6 Discussion

This qualitative case study provides a nuanced framework for implementing PCC, demonstrating that its success hinges on addressing interdependent elements across relational and systemic levels. The co‐design process was particularly effective in surfacing the nuanced tensions – such as those around shared responsibility – that often remain obscured in traditional top‐down implementations. The successful emergence of these critical themes suggests that the retreat's design, underpinned by Goffman's frame analysis, was instrumental in facilitating the necessary ‘frame shifts’ among participants, creating a fertile ground for authentic co‐design.

### 6.1 Shared Responsibility: Navigating the Spectrum of Power and Accountability

Our first theme reveals a critical advancement beyond simply transferring decision‐making authority. The analysis revealed a consensus that shared responsibility is a cornerstone of PCC. This emerged from a clear tension: healthcare practitioners expressed concerns about moral responsibility if patients made choices against medical advice, while patient experts highlighted the burden of being abandoned with difficult decisions.

**Figure 6:**
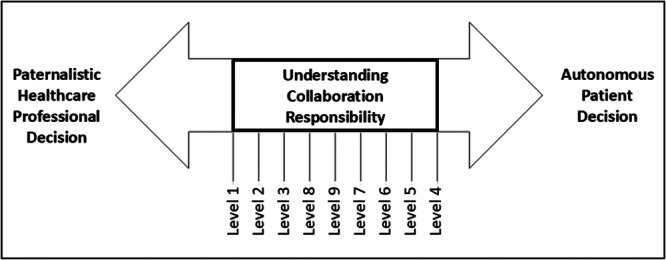
Shared decision‐making is a spectrum. Adapted from El‐Alti L, 2023. Note: (1) Collaboration is on the scale between paternalistic decisions and completely autonomous patient decisions. (2) Explanation of 9 levels as depicted from left to right. Level 1: Patient shares information about herself but HCP makes the decision. Level 2: Patient shares preferences but HCP makes the decision. Level 3: Rational deliberation but HCP makes the decision. Level 8: Rational deliberation followed by joint decision. Level 9: Rational deliberation, conflict, and compromise. Level 7: Rational deliberation but patient makes the decision. Level 6: HCP provides best decision, but patient makes the decision. Level 5: HCP helps patient with preferences, but patient makes the decision. Level 4: HCP shares relevant information, but patient makes the decision.

This finding resonates with but also complicates existing models. El‐Alti conceptualises shared decision‐making as a spectrum between paternalism and patient autonomy (31] [Figure 6). However, our participants’ concerns about blame and abandonment align more closely with Sandman and Munthe's critique that the ideal ‘shared rational deliberative joint decision model’ is often unrealistic due to inherent power imbalances and the clinician's sense of moral responsibility [32]. Consequently, they propose the ‘Professionally Driven Best Interest Compromise’, where the professional strategically influences the patient while still allowing the patient the final decision [32]. This model mirrors the pragmatic negotiation our participants described, acknowledging the clinician's duty while respecting patient choice. The drawback, as one study noted and our participants inferred, is that the manner of presenting information can sway decisions [33], necessitating a balanced account of benefits and risks. Ultimately, as our analysis shows, shared responsibility encompasses a multitude of factors beyond joint decisions, including the patient's best interests, autonomy, the provider's expertise, and the maintenance of a continued therapeutic relationship.

### 6.2 Communication: The Unseen Infrastructure of Care

The second theme positions communication not as a soft skill but as the essential infrastructure of PCC. This theme strongly validates the work of scholars like Hashim, who provides a comprehensive summary of person‐centred communication skills, including expressing empathy, eliciting the patient's agenda, and understanding the patient's perspectives [34]. Our findings confirm that these skills are fundamental to demonstrating empathy and facilitating shared decision‐making [35]. The experience experts at the retreat expressed a strong desire to be heard, feeling their valuable perspectives were not always acknowledged. As our patient co‐author B.P. emphasised, ‘It is human nature to see for themselves ‘evidence’ that PCC is good and how it helps patients/caregivers make better decisions with professionals. What better way than hearing from patients who experienced positive impacts?’ This underscores that effective communication is the primary mechanism through which the evidence for PCC is demonstrated and believed.

Effective rapport‐building is a cornerstone of PCC, directly influencing patient trust and adherence to treatment plans [36]. A range of effective strategies for building rapport have been identified and should be incorporated into healthcare provider training [37]. Therefore, while clinical skills are paramount, it is imperative to systematically integrate these evidence‐based strategies into healthcare education, not as a supplementary skill, but as a foundational component for achieving PCC and improving health outcomes.

### 6.3 Systemic Integration: Building the Pcc Ecosystem

The third theme underscores that individual goodwill is insufficient without a supportive ecosystem. For PCC to be effectively implemented, healthcare providers must be supported by efficient and effective systems that enable smooth transitions, reliable information transfer, and robust collaboration among providers [38].

One potential approach to enhancing PCC is empowering patients with access to their electronic health records (EHRs). Research has shown that such access can provide patients with greater control over their care, improve communication with providers, enhance self‐care capabilities, and foster a sense of empowerment [39]. However, significant questions remain regarding the readiness of our healthcare system to accommodate this level of patient access and control.

‘Leadership support’ was repeatedly cited in discussion notes, moving beyond individual interactions to the organisational structures that govern them. In a white paper prepared for the International Society for Quality in Health Care, Berntsen et al. emphasised the need to embed PCC into the culture of healthcare organisations and make it the norm [40]. They highlighted the necessity of redesigning healthcare systems to make PCC the ‘logical choice’ and to ensure that it is both expected and rewarded. This comprehensive system redesign encompasses multiple domains, including legislation, funding, information systems, education, and research. The ultimate goal is to create a holistic system that supports patient‐defined goals and facilitates a seamless patient journey.

### 6.4 From Abstract Ideals to Concrete Action

The final theme reflects a universal challenge in healthcare improvement: bridging the ‘know‐do’ gap. The concept of person‐centredness is indeed noble, and the vast majority, if not all, agree that PCC is a desirable goal. However, there is a lack of clarity on the concrete steps that can or should be taken to achieve PCC. There is a clear need for actionable steps that both staff and patients can follow. One suggestion was for providers to engage patients with goal setting questions—such as ‘What would you like to achieve at the end of your treatment?’, ‘What is a good life to you?’, and ‘What do you need to do to achieve your goal?’

There is a growing trend of utilising PREMs within the healthcare setting to enhance the quality of care and capture patient perspectives [41]. Using the correct measurements can drive behaviour, as we cannot manage what we do not measure, and these measures can be a simple yet effective way of understanding our patients better. This should also be integrated into the system redesign discussed in the third theme.

Another innovative strategy was to explore new models of care that are inherently person‐centred. One example is the Local Area Coordination and Empowerment (LACE) model [42], which aims to increase the capacity and resilience of individuals, families, communities, and systems by getting to know the whole person and leveraging their strengths. Such models provide concrete strategies that can help facilitate PCC. However, whether the care provided is truly person‐centred ultimately depends on the relationship and interactions between the HCPs and the patients.

## 7 Conclusion

This qualitative case study yields critical insights for implementing PCC in complex healthcare systems. The four themes constructed through our analysis – (1) shared responsibility, (2) communication as foundational, (3) systemic and cultural integration, and (4) actionable implementation – provide a comprehensive framework for advancing PCC.

The retreat's impact extended beyond immediate outputs, fostering sustained engagement and validation of the co‐design process. The patient experience experts were activated to become champions for PCC, as demonstrated by participant and co‐author B.P.'s reflection ‘I use this event as a learning platform to educate other patients and caregivers I encounter… This brings me happiness knowing what I gained is put to good use’.

There are three implications for practice:

- Healthcare systems should invest in communication training that goes beyond information exchange to include relationship‐building and empathy skills.
- Implementation strategies must address both individual clinician‐patient interactions and the systemic enablers that make PCC sustainable.
- Co‐design methodologies that include diverse stakeholders, particularly patient experience experts, can generate more nuanced and implementable PCC approaches.

While this study provides valuable insights into the co‐design of PCC, three limitations should be considered. First, as a qualitative case study of a single retreat, this study does not include a control group or pre‐post quantitative data to measure the long‐term effectiveness of the strategies developed or their impact on clinical outcomes. This design choice was intentional, as the primary aim was to conduct an in‐depth exploration of the *process* of co‐design and to identify key themes and implementation considerations, rather than to test a specific intervention's efficacy.

Second, the findings are based on the perspectives of a purposefully sampled group of stakeholders within a single healthcare cluster. While this approach ensured the inclusion of key voices and achieved thematic saturation, the transferability of the findings to other healthcare contexts with different cultural or structural dynamics may be limited. However, the methodological approach and emergent themes do offer transferable insights for other systems navigating similar transitions to PCC.

Third, as researchers operating from a constructionist stance, we acknowledge that these themes are our interpretive synthesis of the social reality co‐created during the retreat. Our analysis was inevitably shaped by our professional backgrounds within healthcare and our theoretical commitment to viewing PCC as a negotiated, systemic endeavour. Nevertheless, the collaborative and reflexive analytical process, particularly our engagement with experience expert co‐authors and our deliberate exploration of divergent interpretations, was crucial in ensuring these themes remained grounded in the diverse experiences and tensions expressed by the participants themselves.

Despite these limitations, this study offers a rich, empirical account of the practical and philosophical challenges and opportunities in co‐designing a system‐wide approach to PCC, providing a replicable model and a foundational framework for future research.

## Author Contributions

**Xiankun Meng:** writing – original draft, writing – review and editing, data curation, methodology, conceptualisation, formal analysis, visualisation. **Amanda Wei Li Tan:** conceptualisation, writing – original draft, funding acquisition. **Samantha Shi Man Koh:** conceptualisation, writing – original draft, formal analysis, data curation. **Shanice Shi Hui Ng:** writing – original draft, project administration, resources. **Balkhis Puteh:** writing – original draft, writing – review and editing. **Chien Earn Lee:** writing – original draft, writing – review and editing. **Esther Li Ping Lim:** conceptualisation, supervision, writing – original draft, writing – review and editing. **Luke Sher Guan Low:** writing – original draft, writing – review and editing. **Stephanie Swee Hong Teo:** writing – original draft, writing – review and editing. **Andy Gim Hong Sim:** conceptualisation, supervision, writing – original draft, writing – review and editing, methodology, funding acquisition.

## Funding

The authors received no specific funding for this work.

## Ethics Statement

This retreat was conducted as a quality improvement initiative to develop organisational strategy. Per institutional policy, such internal quality improvement activities focusing on service development do not require formal Institutional Review Board review. All participants were informed of the purpose of the retreat and consented to contribute to group activities and outputs. Consent was also obtained from one of the co‐authors of this paper, Ms Balkhis Puteh [B.P.], who was also a participant‐experience expert of the retreat, for her reflections and quotations to be included in this manuscript. Artificial intelligence (ChatGPT) is only used in proofreading of this manuscript.

## Conflicts of Interest

The authors declare no conflicts of interest.

## Supporting information

Supplementary Information.

## Data Availability

The data that support the findings of this study are available from the corresponding author upon reasonable request.
