# Supplementary material for: Trailblazing Person‐Centred Care: Lessons From a Hospital Cluster‐Wide Retreat to Co‐Design Approaches to Person‐Centred Care in Singapore
Source: Health Expect. 2026 Apr 25;29(2):e70594. doi: 10.1111/hex.70594 (PMC13109715; doi:10.1111/hex.70594)
Supplement: Supplementary file 1 — Supplementary Information. [file HEX-29-e70594-s001.docx]

**Consolidated criteria for reporting qualitative studies (COREQ): 32-item checklist**

| **No. Item** | **Guide questions/description** | **Reported on Page #** |
| --- | --- | --- |
| **Domain 1: Research team and reﬂexivity** |  |  |
| *Personal Characteristics* | | |
| 1. Interviewer/facilitator | Which author/s conducted the interview or focus group? | Not a traditional interview study. The "intervention" was a facilitated retreat.  The primary data analysts are named (X.M. and S.K.) in the manuscript. |
| 2. Credentials | What were the researcher’s credentials? E.g. PhD, MD | The researcher’s credentials were reported on the Title page uploaded to Wiley manuscript submission webpage. |
| 3. Occupation | What was their occupation at the time of the study? | The researchers were healthcare practitioners or administrators from different member institutions, but within the same healthcare cluster.  We also had an experience expert as a co-author of this manuscript.  The occupation was reported on the Title page uploaded to Wiley manuscript submission webpage. |
| 4. Gender | Was the researcher male or female? | There are 4 male and 6 female researchers. |
| 5. Experience and training | What experience or training did the researcher have? | All authors have professional healthcare or administrative backgrounds within the SingHealth cluster, except for one patient experience expert co-author (B.P.).  X.M., A.T. and S.K. are healthcare administrators. X.M. and S.K. were former pharmacist and physiotherapist respectively.  C.L. is a medical doctor and deputy CEO of a healthcare organisation.  E.L. is a Chief Allied Health Professional of a healthcare organisation and was a former medical social worker.  L.L. is a medical doctor.  S.T. is Deputy Chief Nurse.  A.S. is a medical social worker with a concurrent appointment as the director of SingHealth Centre for Person-Centred Care. |
| *Relationship with participants* | | |
| 6. Relationship established | Was a relationship established prior to study commencement? | Participants were stakeholders within the same healthcare cluster. Some prior relationship existed with the researchers. |
| 7. Participant knowledge of the interviewer | What did the participants know about the researcher? e.g. personal goals, reasons for doing the research | Participants knew the retreat was organized by SingHealth Center for Person-Centred Care for person-centred co-design.  Specific knowledge of individual researchers was not known. |
| 8. Interviewer characteristics | What characteristics were reported about the interviewer/facilitator? e.g. Bias, assumptions, reasons and interests in the research topic | Facilitators' assumptions/interest in person-centred care are reflected in the retreat design using Goffman's frame analysis to shift perspectives (Section 3, Study Design & Table 1). |

| **Domain 2: study design** |  |  |
| --- | --- | --- |
| *Theoretical framework* | | |
| 9. Methodological orientation and Theory | What methodological orientation was stated to underpin the study? e.g. grounded theory, discourse analysis, ethnography, phenomenology, content analysis | Reflexive Thematic Analysis (Braun & Clarke) is the analytical method. Goffman's Frame Analysis is the conceptual framework for the retreat design (Section 3, Study Design). |
| *Participant selection* | | |
| 10. Sampling | How were participants selected? e.g. purposive, convenience, consecutive, snowball | Participants were selected via purposeful sampling to include key stakeholders (Section 3, Participant Selection and Sampling). |
| 11. Method of approach | How were participants approached? e.g. face-to-face, telephone, mail, email | Participants were approached by “strategic invitation” via email (Section 4, Pre-Design). |
| 12. Sample size | How many participants were in the study? | There were 81 participants (42 healthcare professionals, 26 senior management, 13 experience experts) (Section 3, Participant Selection and Sampling). |
| 13. Non-participation | How many people refused to participate or dropped out? Reasons? | No invited participants refused to participate. Some were unable to attend due to scheduling conflicts. |
| *Setting* | | |
| 14. Setting of data collection | Where was the data collected? e.g. home, clinic, workplace | Data collected during a dedicated, in-person cluster-wide retreat, which was organised in a hotel ballroom. |
| 15. Presence of non-participants | Was anyone else present besides the participants and researchers? | Table facilitators and visual scribe were present (Section 4, Generative Phase). |
| 16. Description of sample | What are the important characteristics of the sample? e.g. demographic data, date | Detailed breakdown: Healthcare Practitioners (52%), Senior Management (32%), Patient/Caregiver Experience Experts (16%) (Section 3, Participant Selection and Sampling; Fig 1). |
| *Data collection* | | |
| 17. Interview guide | Were questions, prompts, guides provided by the authors? Was it pilot tested? | The retreat was structured around four primary framing questions (Section 4, Generative Phase). Questions were not pilot tested, but designed using a theoretical framework. |
| 18. Repeat interviews | Were repeat interviews carried out? If yes, how many? | Not applicable. It was a single retreat event. |
| 19. Audio/visual recording | Did the research use audio or visual recording to collect the data? | The research did not use audio recording.  Visual recording occurred via live-scribed digital artwork (Section 3, The Retreat and Data Collection). |
| 20. Field notes | Were ﬁeld notes made during and/or after the interview or focus group? | Field notes taken by facilitators during the energiser activities, presentations and debate" (Section 3, The Retreat and Data Collection). |
| 21. Duration | What was the duration of the interviews or focus group? | There were no interviews or focus groups conducted. The retreat itself is about 4 hours. |
| 22. Data saturation | Was data saturation discussed? | The related concept of 'thematic sufficiency' (achieved through a sample with high 'information power') is discussed rather than saturation (Section 3, Participant Selection). |
| 23. Transcripts returned | Were transcripts returned to participants for comment and/or correction? | Verbatim transcripts were not created, as the primary data consisted of written notes, visual artifacts, and field notes.  Key outputs (implementation matrix, vision statement) were validated with the retreat participants (Section 3, Data Integration and Validation). |
| **Domain 3: analysis and ﬁndings** |  |  |
| *Data analysis* | | |
| 24. Number of data coders | How many data coders coded the data? | There are two data coders (X.M. and S.K.) (Section 3, Data Analytical Approach). |
| 25. Description of the coding tree | Did authors provide a description of the coding tree? | A coding tree is not provided, but the thematic development process is described, showing how initial codes evolved into final themes (Section 3, Data Analytical Approach & Coder Reflexivity and Handling of Disagreement). |
| 26. Derivation of themes | Were themes identiﬁed in advance or derived from the data? | Themes were inductively derived from the data, informed by broad research objectives (Section 3, Data Analytical Approach). |
| 27. Software | What software, if applicable, was used to manage the data? | Microsoft Word and Excel were used to manage the data (Section 3, Data Analytical Approach). |
| 28. Participant checking | Did participants provide feedback on the ﬁndings? | Member checking by the retreat participants was performed on key outputs (implementation matrix, vision statement) (Section 3, Data Integration and Validation). |
| *Reporting* | | |
| 29. Quotations presented | Were participant quotations presented to illustrate the themes/ﬁndings? Was each quotation identiﬁed? e.g. participant number | Multiple direct quotations are included in the Results (Section 5, Results), identified by speaker type (e.g., "HCP, healthcare professional," "patient experience expert"). |
| 30. Data and ﬁndings consistent | Was there consistency between the data presented and the ﬁndings? | Findings are consistently supported by references to data sources: quotes, world café notes, visual artifacts, field notes (Section 5, Results). |
| 31. Clarity of major themes | Were major themes clearly presented in the ﬁndings? | Four major themes are clearly listed and described in Section 5 (Results). |
| 32. Clarity of minor themes | Is there a description of diverse cases or discussion of minor themes? | The focus is on the four major themes. Divergent interpretations during analysis are discussed as part of the analytical process, revealing tensions (Section 3, Coder Reflexivity and Handling of Disagreement). |
